# Upregulation of hsa_circ_0000977 participates in esophageal squamous cancer progression by sponging miR‐874‐3p

**DOI:** 10.1002/jcla.24458

**Published:** 2022-04-27

**Authors:** Ni Li, Jiacheng Wu, Bingchuan Hu, Hongna Lu, Jianqing Gao, Linwen Zhu, Dawei Zheng

**Affiliations:** ^1^ Department of Cardiothoracic Surgery Lihuili Hospital Affiliated to Ningbo University Ningbo China; ^2^ 12377 Institute of Pharmaceutics College of Pharmaceutical Sciences Zhejiang University Hangzhou China; ^3^ College of Medical Science Ningbo University Ningbo China

**Keywords:** biological function, biomarker, esophageal squamous cancer, hsa_circ_0000977

## Abstract

**Background:**

Esophageal squamous cell carcinoma (ESCC) is one of the most common clinical malignancies of the digestive system, characterized by high mortality but not evident early symptoms. Molecular markers for diagnostic and outcome prediction are urgently needed. Circular RNAs might play essential roles in the progression of ESCC.

**Methods:**

Hsa_circ_0000977 was identified using circRNA microarrays and qRT‐PCR. The diagnostic value of hsa_circ_0000977 was calculated. We also examined in vitro cell functions in ECA109 and TE12 ESCC cells to determine the effect of hsa_circ_0000977. A dual‐luciferase reporter vector validated the binding of hsa_circ_0000977 to miR‐874‐3p.

**Results:**

The top 10 significantly upregulated circRNAs from microarray assays were hsa_circ_0000977, hsa_circ_0006220, hsa_circ_0043278, hsa_circ_0000691, hsa_circ_0000288, hsa_circ_0000367, hsa_circ_0021647, hsa_circ_0006440, hsa_circRNA_405571 and hsa_circRNA_100790, while the top 10 significantly downregulated circRNAs were hsa_circ_0008389, hsa_circ_0089763, hsa_circ_0089762, hsa_circ_0000102, hsa_circ_0001714, hsa_circ_0089761, hsa_circ_0007326, hsa_circ_0001549, hsa_circ_0005133 and hsa_circRNA_405965. Hsa_circ_0000977 was significantly upregulated in ESCC (*p *< 0.01) and had diagnostic value in ESCC. The hsa_circ_0000977 expression level was related to the pT stage and numbers of lymph nodes in ESCC patients. Elevated hsa_circ_0000977 promoted cell proliferation, migration and inhibited apoptosis in ESCC cells. Hsa_circ_0000977 might function as a micro‐RNA sponge to competitively bind miR‐874‐3p.

**Conclusion:**

Disordered hsa_circ_0000977 expression can promote carcinogenesis in ESCC and might serve as a diagnostic biomarker to evaluate the occurrence and development of esophageal cancer.

## Graphical abstract

Hsa_circ_0000977 can serve as a diagnostic biomarker to esophageal squamous cancer, related to the pT stage and numbers of lymph nodes in ESCC patients. Upregulation of hsa_circ_0000977 promoted cell proliferation, migration and inhibited apoptosis in esophageal squamous cancer cells, by sponging miR‐874‐3p.

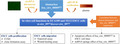

## 1 INTRODUCTION

Esophageal cancer is a common malignant tumor, with more than 400,000 deaths worldwide; there were 600,000 new cases, and the mortality rate was the sixth‐highest worldwide.^1^ Among all esophageal cancer, China accounts for more than 50%, and the mortality rate ranks fourth among tumor deaths.^2^ The histological types can be divided into squamous cell carcinoma (ESCC) and adenocarcinoma. Unlike western countries, in China, 90% is ESCC.^3^ The pathophysiology of esophageal cancer is a multi‐factor process characterized by multigene involvement and mutation and multi‐stage evolution.^4^ Carcinogenesis results from the long‐term combination of environmental and genetic factors.

Traditional treatment methods of ESCC include surgery, radiotherapy, and chemotherapy. The overall efficacy has reached a plateau, with the overall 5‐year survival rate between 15 and 20%. Especially for chemotherapy‐based drugs and systemic therapies, the efficacy has not significantly improved over time. For recurrent or advanced metastatic ESCC, the objective response rate of first‐line chemotherapy was 30%–50%, with a median progression‐free survival (PFS) of 4–6 months and median overall survival (OS) of 9–12 months.^5^, ^6^ The objective response rate of second‐line chemotherapy was 6%–34%, with a median PFS of 2–4.5 months and a median OS of 5–8.1 months.^7^ Many clinical trials of targeted molecular treatment for ESCC have failed. However, the rise of immunotherapy broke the deadlock. In 2019, the phase III clinical study keynote‐181 released the results. For the second‐line treatment of esophageal cancer with PD‐L1 (programmed cell death 1 ligand 1) CPS (combined positive score) ≥10, pembrolizumab was significantly better than chemotherapy. Although immunotherapy can be referred to as the light of hope for treatment of ESCC, the courses currently used in immunotherapy are not clear, and there are no biomarkers to evaluate efficacy. Immunotherapy carries a high incidence of adverse reactions that restrict its use. Therefore, it is crucial to explore the mechanisms of ESCC to identify effective therapeutic targets and identify biomarkers to assess efficacy.

Circular RNA (circRNA) is endogenous RNAs divided into noncoding circRNAs and coding circRNAs, which can differentially expressed in many diseases.^8^ Unlike linear RNA, the structure of circRNA is stable and not susceptible to degradation by nuclease.^9^ However, recent studies showed that circRNA acts as a micro‐RNA (miRNA) sponge to regulate gene expression in several ways.^10^ Many studies demonstrate that miRNA plays an essential regulatory role in tumorigenesis and development.^11^, ^12^ Based on the function of circRNA to sponge adsorb miRNA, circRNA plays a minor role in regulating tumorigenesis and development.^13^

Hsa_circ_0000977 has not yet been associated with esophageal cancer. Therefore, we performed the following study to provide a basis for the diagnosis and treatment of ESCC. We identified hsa_circ_0000977 as a circRNA that was significantly upregulated in ESCC plasma, using circRNA microarray profiling, to further expand the sample validation by quantitative real‐time reverse transcription‐polymerase chain reaction (qRT‐PCR). Bioinformation prediction for the miRNA possibly bind was made that hsa_circ_0000977 can be combined through miR‐874‐3p. In vivo cell function experiments including ECA109 and TE12 ESCC cells show that hsa_circ_0000977 can promote the proliferation and migration of ESCC cells by sponging miR‐874‐3p, which can provide new clues for the diagnosis and treatment of esophageal cancer.

## 2 MATERIALS AND METHODS

### 2.1 Patient samples and cell lines

Twenty‐five plasma pairs were obtained from ESCC patients who underwent surgery at Lihuili Hospital Affiliated to Ningbo University (Ningbo, China), between June 2020 and September 2021. The patients included 23 males and two females. The age range was 44 to 80 years, with a mean age of 62.72 years. Three pairs of ESCC samples and controls were used for circRNA array profiling. The remaining 25 pairs of ESCC samples and controls were used for validation by using quantitative real‐time reverse transcription‐polymerase chain reaction (qRT‐PCR). The inclusion criteria were as follows: Group 1: diagnosis of ESCC (>18 years old) by gastroscopy and pathology, without radiotherapy, chemotherapy, or biological therapy. Group 2: benign esophageal lesion. Four patients were diagnosed with benign esophageal tumors by gastroscopy. Esophageal leiomyoma (ESL) was not treated with radiotherapy, chemotherapy, or biological therapy. Exclusion criteria were as follows: (i) malignant tumors other than ESCC; (ii) previous radiotherapy, chemotherapy, and biological therapy, and no radiotherapy, chemotherapy, or targeted therapy before surgery; and (iii) other serious diseases. We collated patient‐related clinical data and risk factors, including gender, age, family history, smoking history, tumor T grade, lymph node N stage, distant metastasis, and clinical for all post‐diagnosis and pretreatment blood samples stages (Table 1). All experiments were carried out following relevant guidelines and regulations of Lihuili Hospital. The Ethics Committee approved the study, and all patients provided written informed consent. The ESCC cell lines (TE12 and ECA109) were obtained from the Chinese Academy of Sciences (Shanghai, China).

**TABLE 1:** Correlation between hsa_circ_0000977 expression and clinical parameters in ESCC

Characteristics		Numbers	Hsa_circ_0000977 expression			
			high	low	χ^2^	P
Gender	Male	23	11	12	0.003484	0.9529
	Female	2	1	1		
Age(years)	>60	15	6	9	0.9615	0.3268
	≤60	10	6	4		
Tumor location	Upper	4	1	3	1.453	0.4835
	Middle	11	5	6		
	Lower	10	6	4		
Smoking	No	8	5	3	0.9910	0.3195
	Yes	17	7	10		
Drinking	No	11	4	7	1.066	0.3019
	Yes	14	8	6		
Family history	No	24	12	12	0.9615	0.3268
	Yes	1	0	1		
Tumor location	>3cm	15	7	8	0.0267	0.8702
	≤3cm	10	5	5		
Differentiation	High	3	0	3	3.147	0.0761
	Moderate& Poor	10	12	10		
pT stage	pT2	1	0	1	7.287	0.0262*
	pT3	19	12	7		
	pT4	5	0	5		
pN stage	pN0	3	3	0	4.911	0.1785
	pN1	5	3	2		
	pN2	7	3	4		
	pN3	10	3	7		
pTNM stage	I‐II	4	3	1	1.391	0.2383
	III‐IV	21	9	12		
Total lymph node dissection(n)	≥15	6	5	1	3.949	0.0469*
	<15	19	7	12		

### 2.2 CircRNA microarray

Three ESC plasma pairs and controls were used for circRNA microarrays. NanoDrop ND‐1000 was used for total RNA quantification. CircRNA was enriched, amplified, and transcribed into fluorescent cRNA. Agilent Feature Extraction software (version 11.0.1.1) was used to analyze acquired array images. Data analysis was done using R software limma package. The circRNA array data were as previously described.^10^ Differentially expressed circRNAs changes were identified with a fold change of >2.0 and *p*‐value <0.05 as significant.

### 2.3 Extraction of total RNA from the plasma and qRT‐PCR

We centrifuged 2–4 ml of peripheral blood at 3000 rpm for 15 min. The upper plasma was stored at −80°C. Total RNA extraction using the TRIzol LS reagent (Invitrogen) and reverse transcription were performed as previously described.^10^ ABI 7500 System (Applied Biosystems, USA). GAPDH was used as the reference gene. All the experiments were performed in triplicate, and the 2^−ΔΔCT^ value was used to represent circRNAs relative expression levels. The primer sequences are listed in Table S1.

### 2.4 The diagnostic value analysis

Diagnostic value was calculated according to the hsa_circ_0000977 relative expressions in plasma combined with clinicopathological factors. The relationship between the clinicopathological factors and hsa_circ_0000977 expression was also analyzed. Receiver operating characteristic (ROC) curves were drawn to calculate the area under the curve (AUC) and determine the diagnostic value of hsa_circ_0000977 in esophageal cancer.

### 2.5 Cell culture and transfection

ECA109 or TE12 cells were cultured in RPMI 1640  DMEM ormedia respectively containing 10% FBS(fetal bovine serum), with 50 U/ml streptomycin, and 50 U/ml penicillin. Overexpression and silencing of hsa_circ_0000977 were performed in esophageal cancer cell lines. Recombinant plasmid for upregulated hsa_circ_0000977 was constructed and ligated to pcd5 with the target gene fragment using double digestion. The overexpression and silencing efficiency of hsa_circ_0000977 in two cells were determined using qRT‐PCR. Vectors for overexpression of hsa_circ_0000977, si‐hsa_circ_0000977, and negative controls were obtained from the GenePharma Company (Shanghai, China). RNA oligonucleotide sequences are listed in Table S2. The hsa_circ_0000977 siRNA and overexpression plasmids were transfected into ECA109 or TE12 cells using Lipofectamine 2000 (Invitrogen) for 48 hours. The transfection efficiency was measured using qRT‐PCR.

### 2.6 Cell proliferation

The Esophageal cancer cell proliferation was evaluated by CCK‐8 and colony formation assays, which are performed as previously.^10^ 5 × 10^3^ cells per well of transfected cells with different vectors were seeded into 96‐well plates at different time points (1day, 2day, 3day, 4day, and 5 day) 10 μl of CCK‐8 reagent (Dojindo, Japan) was added to each well, and incubated at 37°C for 1–3 h. Finally, the absorbance was measured at 450 nm.

### 2.7 Clone formation assay detection

Esophageal cancer cell lines with overexpressed or silenced hsa_circ_0000977 were grown for 24 h. Cells were trypsinized and gently aspirated into a sterile straw. Six‐well plates were incubated for 10 to 15 days. The time was adjusted by visually observing for clonal spots when clonal spots appeared on visual inspection. Each well was fixed with 1 ml 4% paraformaldehyde solution in clonal spots for 30 min. Paraformaldehyde was removed, and clonal spots were stained with 1 ml of 0.1% crystal violet for 30 min per well. The crystal violet dye was removed, and cells were gently rinsed with tap water, dried, photographed, and statistically analyzed.

### 2.8 Transwell assay

Twenty‐four‐well Transwell chambers (Corning) were used for cell migration measurement. The transfected cells were harvested and resuspended in a serum‐free medium. Two hundred microliters of the cells was inoculated into the upper chamber and 500 µl of medium containing 20% fetal bovine were added to the lower chamber for 24h incubation. Finally, the cells were fixed with 4% paraformaldehyde and stained with 0.1% crystal violet. All experiments were performed in triplicate.

### 2.9 Apoptosis assay

Transfected ECA109 and TE12 cells were collected and washed with phosphate‐buffered saline and then analyzed with an Annexin V: PI apoptosis detection kit (BD Bioscience, USA). The apoptosis rate was measured using flow cytometry (FACS Caliber BD, USA).

### 2.10 Dual‐luciferase reporter assay

The day before transfection, the experimental cells were seeded into 24‐well plates at 11^4^ cells per well. The pRL‐SV40 were co‐transfected with pGL‐SV40 and pGL3‐control and pGL3‐hsa_circ_0000977‐WT and pGL‐3‐hsa_circ_0000977‐MUT, respectively. Changes in luciferase activity were analyzed at 24 h after transfection.

### 2.11 Statistical analysis

Statistical analyses were performed using GraphPad Prism v6.0 (GraphPad Software Inc., USA). Data were expressed as mean ± standard deviation. *p *< 0.05 was considered significant.

## 3 RESULTS

### 3.1 CircRNAs expression profiles

**FIGURE 1:**
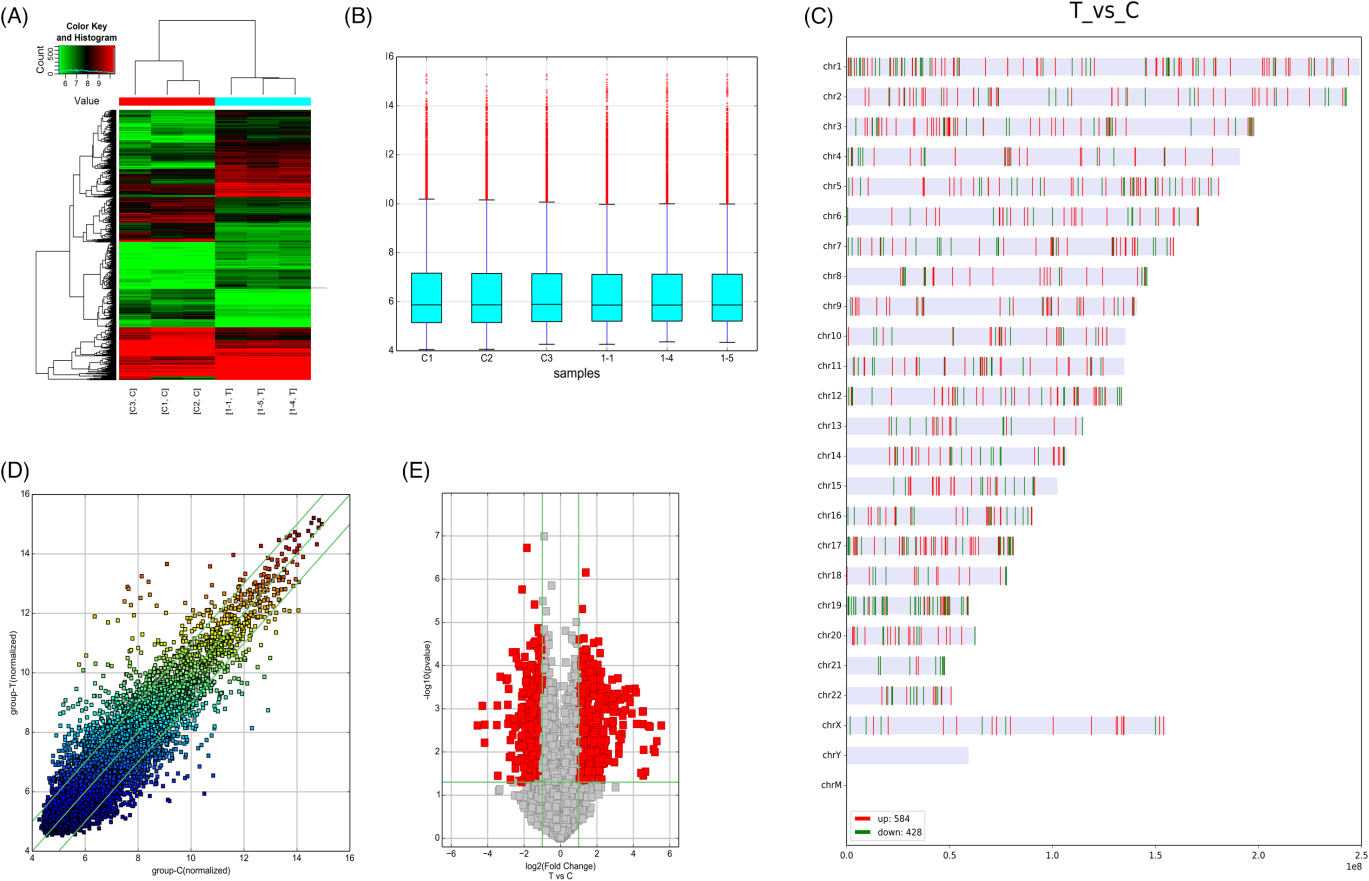
CircRNAs microarray assay and expression profiles in ESCC patients and healthy controls. (A) Hierarchical clustering revealing several circRNAs expressed in three pairs of ESC patients and healthy controls. (B) Box plots showing that the samples had comparable intensities.(C) Chromosomal distribution showing that most differentially expressed circRNAs were transcribed from chr1, chr2, chr3, chr4, chr5, chr6, chr7, chrX. of (D) Scatter plots‐CircRNAs (E) Volcano plots‐CircRNAs showed differentially expressed circRNAs between ESCC and control groups.

CircRNA expression profiles were analyzed in three esophageal cancer patients and three healthy control using microarray analysis. A total of 11381 circRNAs were identified and significantly differently expressed between ESCC patients and matched healthy controls. Of these, 5705 circRNAs (50.13%) were upregulated and 5676 (49.87%) were downregulated; 584 circRNAs were upregulated (*p *< 0.05) and 428 were downregulated significantly (*p *< 0.05). Hierarchical clustering analysis (Figure 1A), box plot (Figure 1B), chromosomal distribution (Figure 1C), scatter plots‐circRNAs (Figure 1D), and volcano plots‐circRNAs (Figure 1E) demonstrated differential circRNA expression between ESCC groups and control groups. The top 10 upregulated circRNAs from microarray assays were listed as follows, including hsa_circ_0000977, hsa_circ_0006220, hsa_circ_0043278, hsa_circ_0000691, hsa_circ_0000288, hsa_circ_0000367, hsa_circ_0021647, hsa_circ_0006440, hsa_circRNA_405571, and hsa_circRNA_100790. Meanwhile the top 10 downregulated circRNAs were hsa_circ_0008389, hsa_circ_0089763, hsa_circ_0089762, hsa_circ_0000102, hsa_circ_0001714, hsa_circ_0089761, hsa_circ_0007326, hsa_circ_0001549, hsa_circ_0005133, and hsa_circRNA_405965 (Table S3).

### 3.2 Characterization and specific primers of hsa_ circ_0000977

**FIGURE 2:**
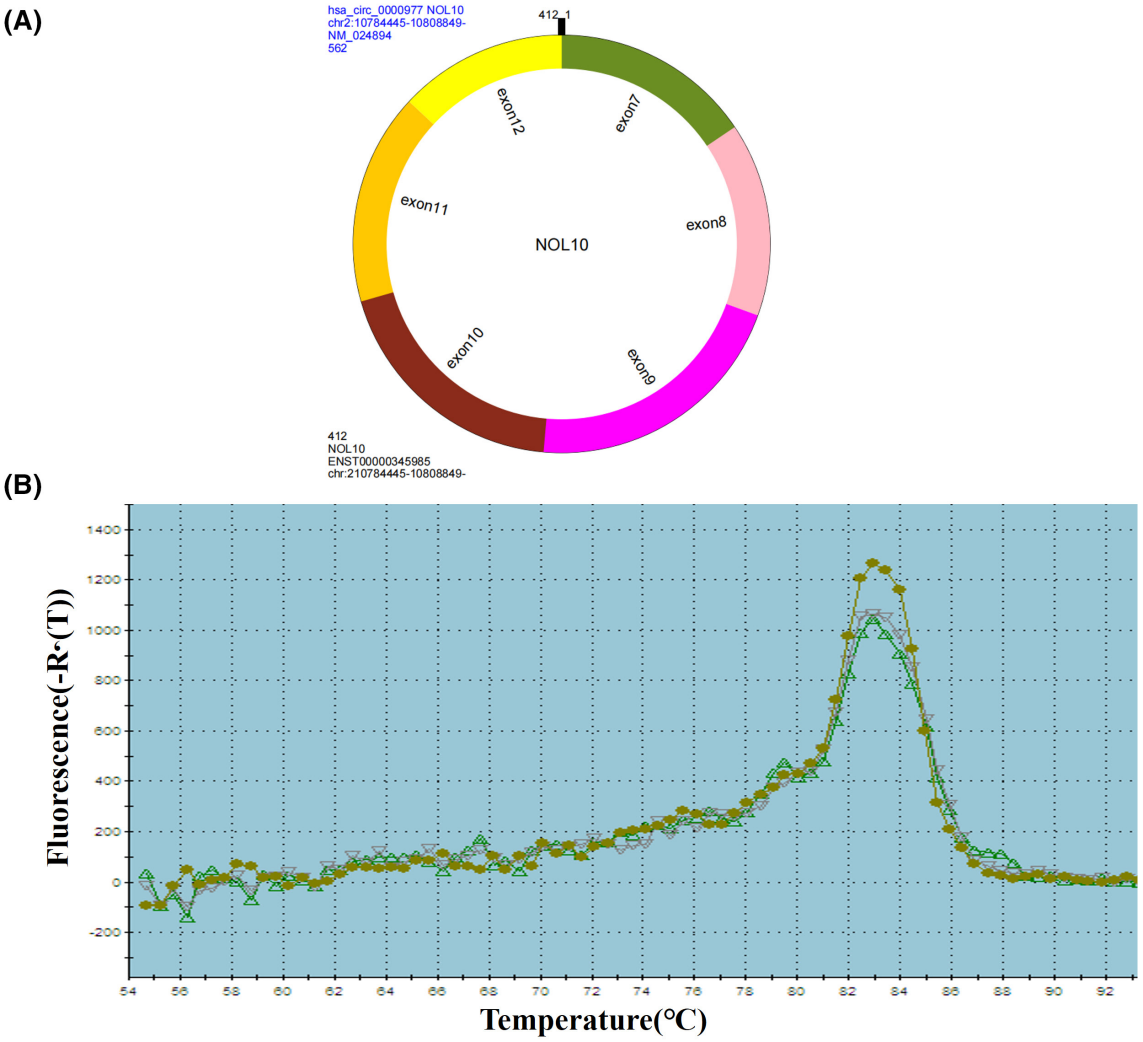
Amplification of hsa_circ_0000977. (A) hsa_circ_0000977 is an exonic circRNA. (B) Melting curve analysis of the qRT‐PCR products of hsa_circ_0000977, three representative samples are revealed.

As shown in Figure 2A, the hsa_circ_0000977 is composed of exon 7 to exon 12. The amplified product reached only one peak (Figure 2B). From the microarray results, hsa_circ_0000977 was selected from upregulated circRNAs as a candidate clinically relevant biomarker according to 46.37‐fold change. The *p*‐value was 0.00244803. The five predicted target miRNAs (miRNA‐874‐3p, miRNA‐767‐5p, miRNA‐452‐5p, miRNA‐281‐1‐3p, and miRNA‐281‐2‐3p) were associated with tumor progression. Based on these criteria, hsa_circ_0000977 was selected for further validation.

### 3.3 Hsa_circRNA_0000977 is a diagnostic biomarker in ESCC

**FIGURE 3:**
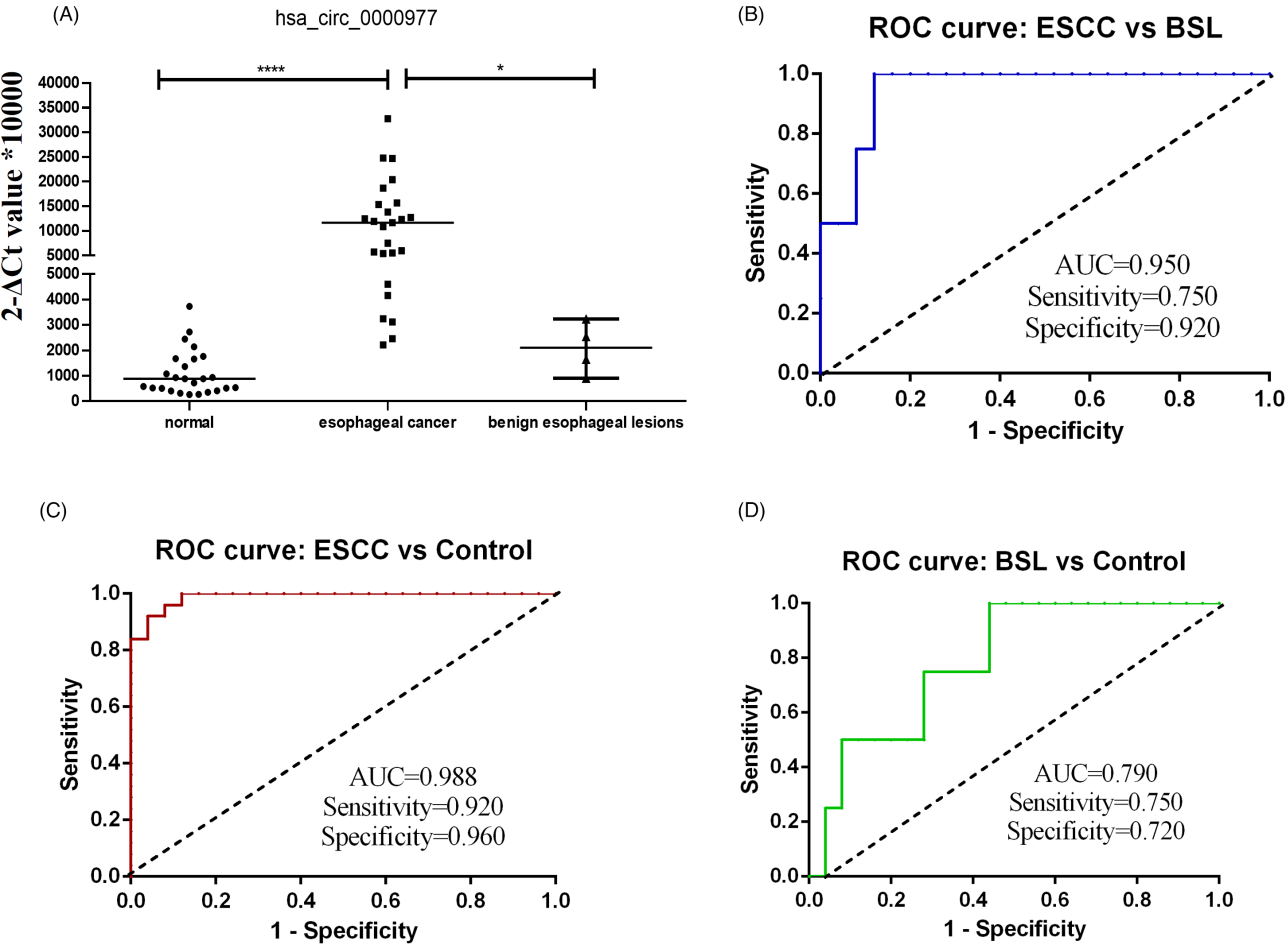
Hsa_circ_0000977 expression in ESCC plasma samples and their diagnostic values. (A) Expression levels of hsa_circ_0000977 in the ESCC were significantly higher than those of ESL group and the normal control group (*n *= 25). (B) AUC of the ESCC group (*n *= 25) compared with the normal control group (*n *= 25). (C) AUC of the ESCC group (*n *= 25) compared with the ESL group (*n *= 4). (D) AUC of the ESL group (*n *= 25) compared with the normal control group (*n *= 25). AUC: the area under the curve, *p < 0.05, ****p < 0.0001.

Based on qRT‐PCR validation, hsa_circ_0000977 was higher in the plasma of ESCC patients than control group. The expression levels of hsa_circ_0000977 in plasma samples of 25 ESCC patients, 4 benign esophageal lesions (BSL) patients, and 25 normal controls were determined. Levels of hsa_circ_0000977 were significantly higher in ESCC patients than in normal controls. The expression of hsa_circ_0000977 increased significantly, ESCC group (*n* = 25) vs. control group (*n* = 25), *p* < 0.0001; ESCC group (*n* = 25) vs. BSL group (*n* = 4), *p *= 0.0281; but no significant difference between BSL group and control group (*p *= 0.0541), which is suggesting that the upregulation of hsa_circ_0000977 was positively associated with esophageal malignancy (Figure 3A). The qPCR validation result is also consistent with the circRNA microarray analysis results. Then, ROC curve analysis was drawn to calculate the diagnostic values of hsa_circ_0000977 for ESCC (Figure 3B–D). Compared with the 25 control samples, AUC of the ESCC group (*n* = 25) was 0.9888 (95% CI: 0.9695 to 1.008, *p *< 0.0001). The sensitivity was 0.92, and specificity was 0.96 (Figure 3C). While compared with the ESL group (*n *= 4), AUC of the ESCC group (*n *= 25) was 0.9500 (95% CI: 0.8686 to 1.031, *p *= 0.004), the sensitivity was 0.75 and specificity was 0.92 (Figure 3B). AUC of the ESL group (*n *= 4) compared with the normal control group (*n *= 25) was 0.79 (95% CI: 0.5917 to 0.9883, *p *= 0.0667), the sensitivity was 0.75, and specificity was 0.72 (Figure 3D).

### 3.4 Correlation between clinical parameters and hsa_circ_0000977 expression in ESCC

Chi‐square tests analyzed the correlation between clinical parameters and hsa_circ_0000977 expression, such as gender, age, risk factors, and pathological TNM stage. There is a significant difference among different pT stages among pT2, pT3, pT4 groups, *p *= 0.0262, and the number of positive lymph node dissections between ≥15 and <15 groups shows significant differences = 0.0469 (Table 1). However, there are no significant differences among other clinical parameters, including age, gender, smoking, drinking, tumor location positions, differentiation, pN, and pTNM stage.

### 3.5 Hsa_circ_0000977 promotes the proliferation of ECA109 and TE12 cells

**FIGURE 4:**
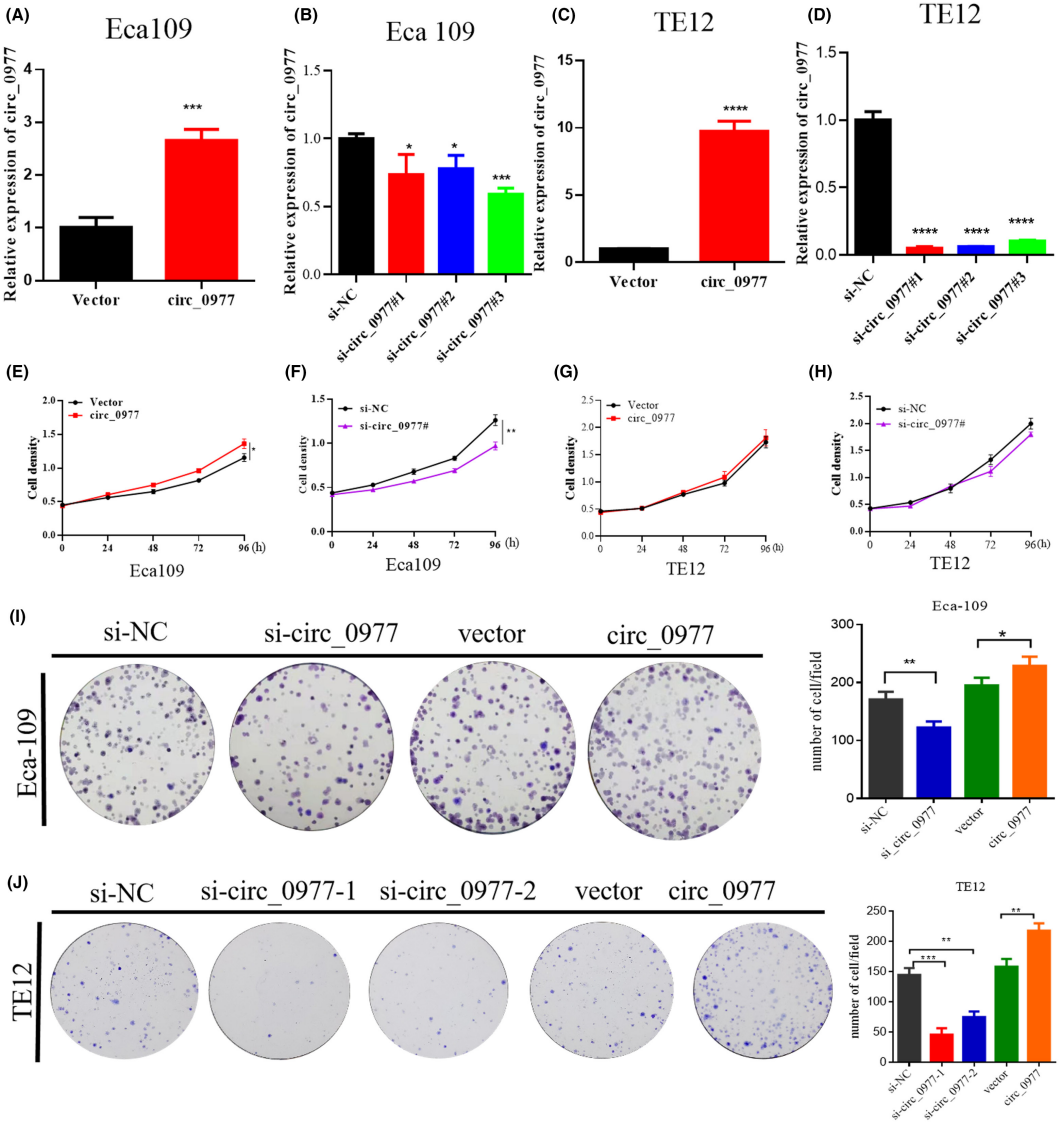
Effects of up and downregulation of hsa_circ_0000977 on ECA109 and TE12 proliferation. (A,B) siRNA upregulation effect and downregulation effect of hsa_circ_0000977 in ECA109 cells. (C,D) siRNA upregulation effect and siRNA downregulation effect in TE cells. Three si‐circ_0000977 were selected. (E, F) Grow curve of ECA109 cells following upregulation or downregulation of hsa_circ_0000977. (G,H) Grow curve of TE cells following upregulation or downregulation of hsa_circ_0000977. (I) Colony formation assays after hsa_circ_0000977 knockdown or overexpression in ECA109 cells. (J) Colony formation assays to measure proliferation after hsa_circ_0000977 knockdown or overexpression in TE12 cells.NC, negative control; *n* = 3,*p < 0.05, **p < 0.01,***p < 0.001.

In vitro cell functions in ECA109 and TE12 ESCC cells were performed to study the function of hsa_circ_0000977 in ESCC progression. The relative expression hsa_circ_0000977 was effectively silenced by si_circ_0977#1, 0977#2, 0977#3 (three si‐circs) and significantly upregulated by overexpression (circ_0977) in both ECA109 and TE12 cells (Figure 4A–D). A CCK‐8 assay showed that si‐circRNA suppressed cell growth (Figure 4F), and circ_0977 promoted cell growth in both ESCC cells (Figure 4E) in ECA109, while no significant difference in TE12 cell (Figure 4G,H). At the same time, si‐circ_0977 can inhibit proliferation, while over‐circ_0977 promoted from colony formation assay results in ECA109 cells (Figure 4I) and TE12 cells (Figure 4J).

### 3.6 Hsa_circ_0000977 promotes the migration of ECA109 and TE12 cells and inhibits their apoptosis

**FIGURE 5:**
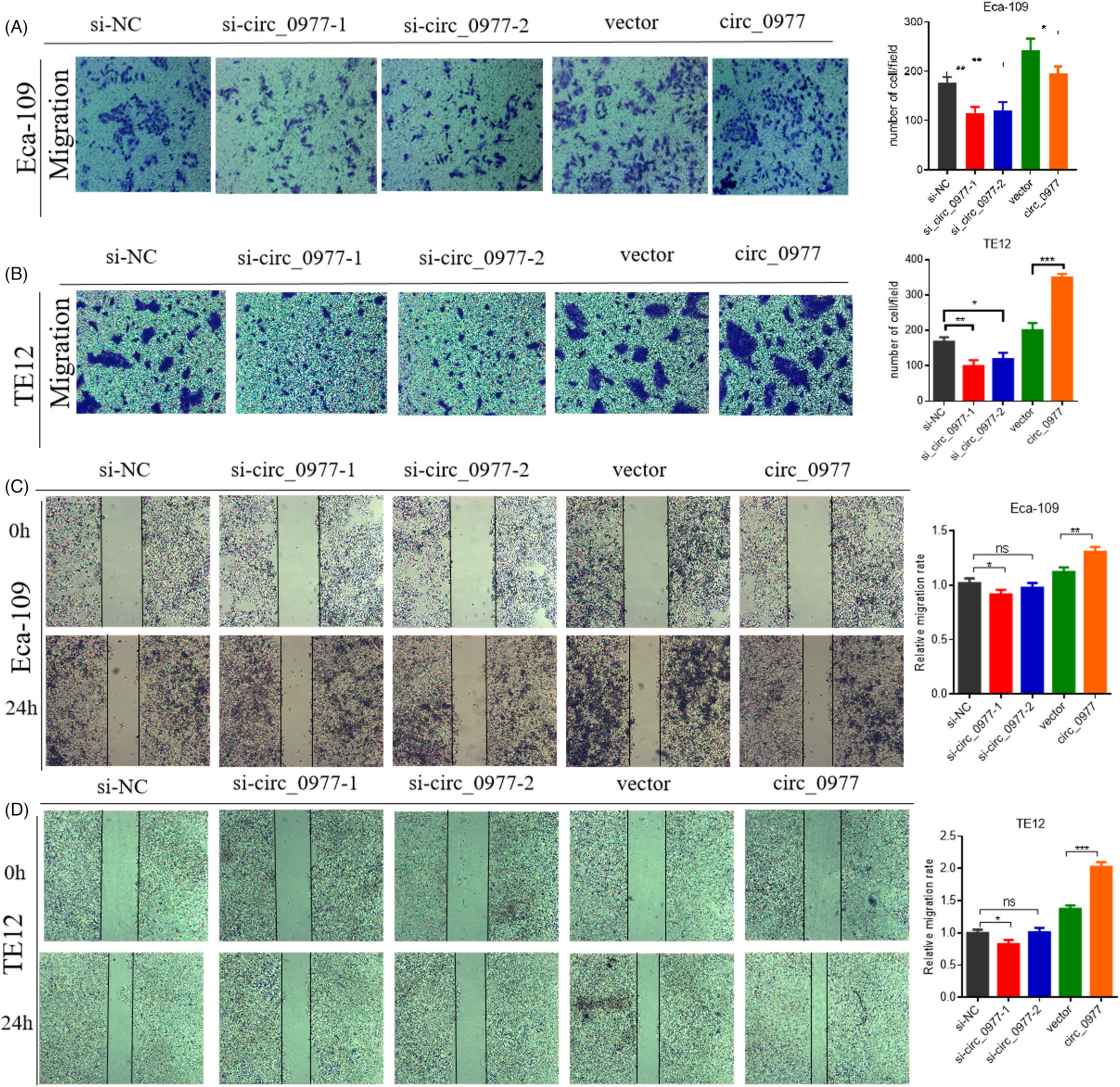
Effects of hsa_circ_0000977 on ESCC cell migration. (A) Transwell assays to measure cell migration of ECA109 cells. (B) Transwell assays to measure cell migration of TE‐12 cells. (C) Wound‐healing effects of hsa_circ_0000977 in ECA109 cells. (D) Wound‐healing assays in TE12 cells. NC, negative control; si, knockdown; *n* = 3, **p < 0.01, *p < 0.05.

**FIGURE 6:**
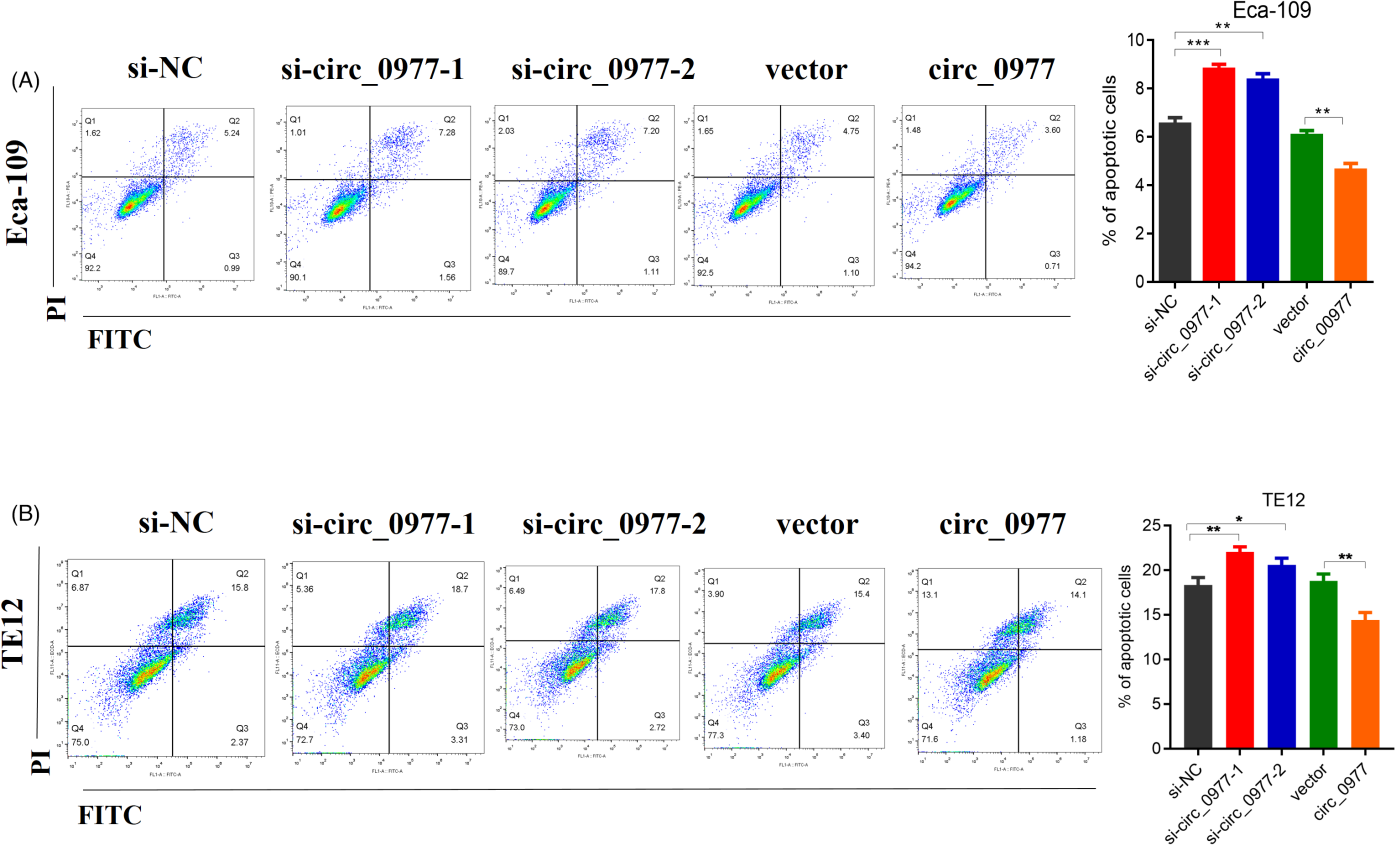
Apoptosis effects of hsa_circ_0000977 in ESCC cell lines (measured using flow cytometry). (A) The apoptosis rate of ECA109 following downregulation and upregulation of hsa_circ_0000977. (B) The apoptosis rate of TE12 following downregulation and upregulation of hsa_circ_0000977. *n* = 3, *p < 0.05, ***p < 0.001.

The Transwell assays showed that si‐circ_0977 significantly decreased migration, over‐circ_0977 increased migration in ECA109 (Figure 5A) and TE12 cells (Figure 5B). It is also demonstrated that types of si‐circ_0977 can significantly decrease migration, but overexpression (circ_0977) increased migration in ECA109 (Figure 5C) and TE12 cells (Figure 5D) as seen in the wound healing assays. Furthermore, si‐circ_0977 increased the numbers of apoptotic cells in ECA109 cells (Figure 6A) and TE12 cells significantly (Figure 6B), while decreased the number of apoptotic cells with over‐circ_0977. From these results, it is suggested that hsa_circ_0000977 inhibited apoptosis in ESCC cell lines.

### 3.7 **Hsa_circ_0000977** **serves as a sponge for miR‐487‐5p and bioinformation prediction of the target genes**

**FIGURE 7:**
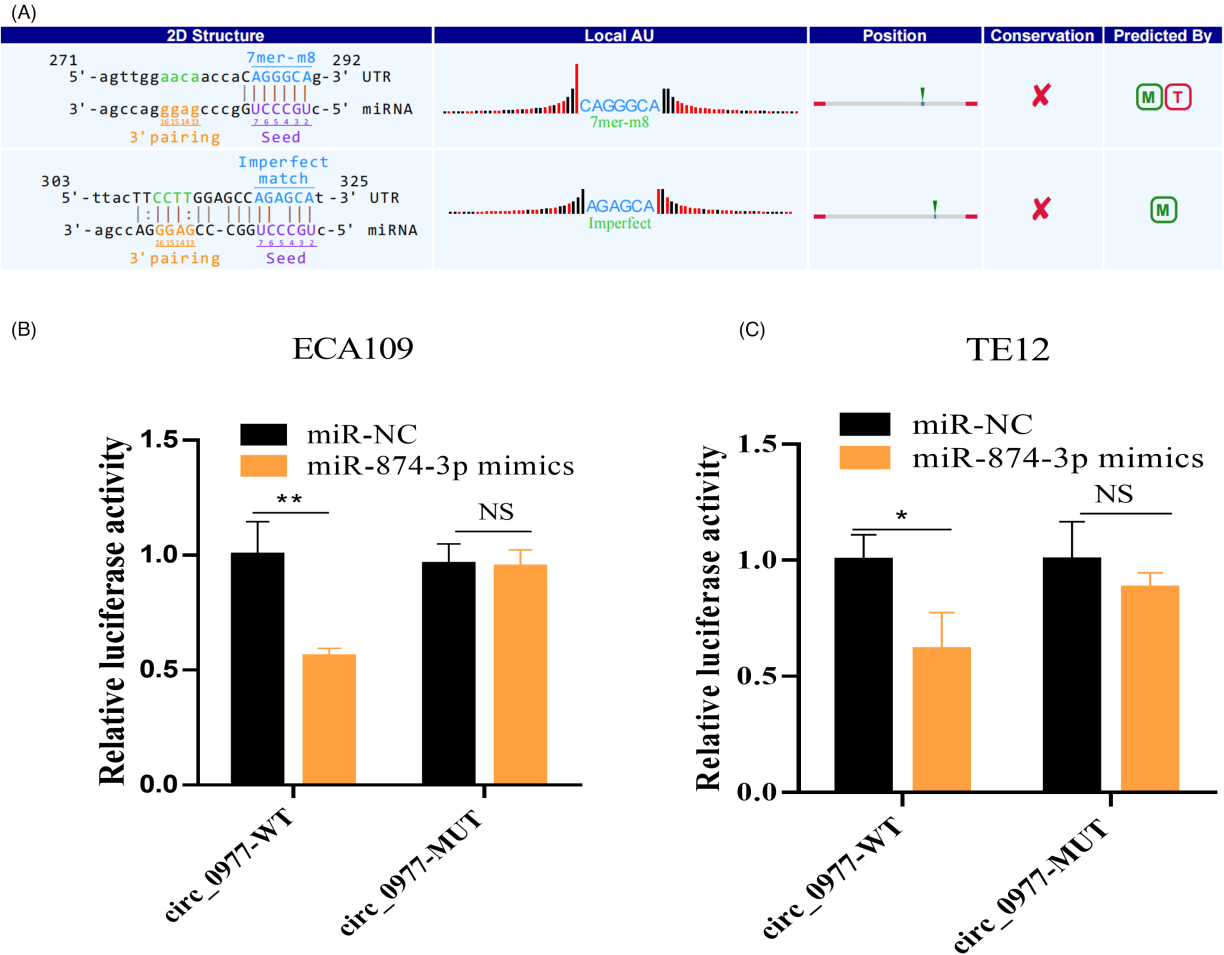
miRNA sponge effect of hsa_circ_000977. (A) Bioinformatics prediction of the combination of hsa_circ_000977 and miR‐874‐3p. (B) Dual‐luciferase reporter vector validating the binding of hsa_circ_000977 to miR‐874‐3p in ECA109. (C) Dual‐luciferase reporter vector validating the binding of hsa_circ_000977 to miR‐874‐3p in TE12.

CircRNA interaction software was used for the prediction of the miRNAs bounding to hsa_ circ_0000977. A combination of hsa_circ_0000977 and miR‐487‐5p was found by bioinformatics prediction (Figure 7A). The binding sites for hsa_circ_0000977 and miR‐487‐5p were verified by dual‐luciferase reporter gene experiments in ECA109 and TE12 cells (Figure 7B, C). To further explore how the hsa_circ_0000977 sponge affects the downstream pathway after miR‐874‐3p, the target genes of miR‐874‐3p were predicted through the miRNA databases of TargetScan, Starbase (ENCORI) using two different algorithms, finally screened a total of 1145 (324, 275, and 546 respectively) target genes using various algorithms. After interactive analysis of the above three groups of predicted target genes, three highly reliable target genes were screened, namely TP53INP2 (tumor protein p53 inducible nuclear protein 2), OSTM1 (osteoclastogenesis associated transmembrane protein 1), and KMT2D (lysine methyltransferase 2D) (Fig. S1), which could be selected for further mechanism study.

## 4 DISCUSSION

The risk factors for esophageal cancer mainly include alcohol consumption, smoking, trace element deficiency, hot eating, male sex, obesity, also head and neck cancer history. Nevertheless, individuals exposed to the same environment and identical lifestyle habits have different incidences of esophageal cancer, suggesting that other factors (including genetic susceptibilities) add to the risk of esophageal cancer.^14^, ^15^ Several studies reported that the regulatory axis of circRNA‐miRNA is associated with the pathophysiology of various diseases, including esophageal cancer, by regulating gene transcription and translating proteins.^16^, ^17^ The regulatory axis of circRNA‐miRNA can participate in disease progression.^18^ Compared to other competing endogenous RNA, its miRNA sponge effect is more persistent due to the structural stability of circRNA.^19^, ^20^ Zhang et al. found a novel hsa_circRNA6448‐14. This circRNA can influence ESCC progress by binding miR‐455‐3.^16^ In our study, 11381 circRNAs were screened to be significantly differentially regulated in ESCC by microarrays, which might affect specific biological processes. We found the proportions of upregulated and downregulated circRNAs were 50.13% and 49.87%, respectively. Both microarray assay and qRT‐PCR significantly upregulated the expression of hsa_circ_0000977. This study used the regulatory axis of circRNA‐miRNA to explore the molecular mechanism of esophageal cancer proliferation by hsa_circ_0000977.

The qRT‐PCR validation demonstrated the upregulation of hsa_circ_0000977 in ESCC. To our knowledge, hsa_circ_000977 had not been previously reported as an oncogene in ESCC. Many circRNAs exist as single or multiple exons.^21^ Hsa_circ_0000977 is an exonic circRNA that originates from exon 7 to 12 of the NOL10 gene located on chromosome 2. We tried to verify the correlation between the expression of hsa_circ_0000977 in ESCC plasma and clinical parameters. We found that pT stage and the number of positive dissected lymph node positively correlated with hsa_circ_0000977 expression in plasma. The pT stage and the pathology results of dissected lymph node are crucial for assessing the severity of esophageal cancer. Clinical retrospective studies showed that higher pT stage and higher number of positive lymph nodes correlate with shorter OS and DFS. The other clinical information was not shown to be related to the expression of hsa_circ_0000977, maybe because of the small sample size, which needs further research in the future. From our findings, hsa_circ_0000977 is associated with esophageal cancer staging and be used as a biomarker to predict the outcomes of esophageal cancer.

In early stage ESCC, endoscopy is considered the gold standard for its diagnosis. To distinguish between high‐grade dysplasia and cancer, it is reported that the sensitivity and specificity of white‐light endoscope is 62% and 79%, respectively.^22^ When using Lugol chromoendoscopy, a much higher sensitivity of 96% and meantime a slight reduction of specificity of 63% were obtained.^23^ Because endoscopy is invasive and its effectiveness depends on the experience of the operator, recently quite a number of techniques for esophageal cytology developed such as inflatable balls and sponges. However, these techniques cannot satisfy both sensitivity and specificity, therefore unsuitable for mass screening. The hsa_circ_0000977 of this study showed a better sensitivity and specificity. Compared to endoscopic diagnosis, circRNA detection is noninvasive, also with higher economic benefits, simpler operation, and patients might have better adherence to examination.

In vitro cell functions demonstrated that hsa_circ_0000977 participated in ESCC proliferation, migration, and inhibited apoptosis, suggesting that hsa_circ_0000977 promotes the progression of ESCC. Subsequently, it is found that hsa_circ_0000977 can bind to miR‐874‐3p in ECA109 and TE12 using a dual‐luciferase reporter vector.

To explore how the hsa_circ_0000977 sponge affects the downstream pathway after miR‐874‐3p,^24^ we predicted the target miR‐874‐3p using Targetscan,^25^ TarBase,^26^ and ENCORI.^27^ After interactive analysis of these groups of predicted target genes, three highly reliable target genes were identified (TP53INP2, OSTM1, and KMT2D). The protein encoded by TP53INP2 gene promotes autophagy, which is critical for the formation and processing of autophagosomes. TP53INP increases the sensitivity of cells to apoptosis induced by death receptor ligands. Zhou et al.^28^ found that the migration and epithelial‐to‐mesenchymal transition (EMT) of cancer cells was associated with decreased TP53INP2 inhibition. This finding suggests that when miR‐874‐3p loses the inhibition of TP53INP2 expression after being sponged, it may promote TP53INP to enhance the migration, invasion, and EMT of esophageal cancer cells. KMT2D methylates lysine 4 in histone H3 in several cancers.^29^, ^30^, ^31^ Our findings suggest that hsa_circ_0000977 may also promote the progression of esophageal cancer through the hsa_circ_0000977/miR‐874‐3p/KMT2D axis. However, there is a lack of reports on OSTIM in tumor‐related mechanisms.

We also performed GO/KEGG enrichment analysis on 1145 prediction target genes screened from the database.^32^ GO analysis revealed that most target genes could be involved in biological processes such as myeloid cell differentiation, protein autophosphorylation, regulation of protein autophosphorylation, and cell growth. KEGG enrichment analysis showed that the target genes are enriched in non‐small‐cell lung cancer, cell senescence, the Hippo signaling pathway, and the chronic myeloid leukemia pathway, shown in Table S4. The molecular functions and pathways have been reported in several cancers. For example, dysregulated Hippo pathway and Yap/TAZ‐TEAD activity are associated with cancer and might participate in organ development and immune regulation. This pathway might serve as an essential target of cancer treatment intervention. In ESCC, hsa_circ_0000977 binds miR‐874‐3p as a molecular sponge, possibly relieving the inhibition of miR‐874‐3p of downstream target genes. The activated target genes promote ESCC progression via these molecular functions and modulate the associated enrichment pathways. However, this study has several limitations, for example, the clinical sample size was limited and experimental verification of biological information analysis needed to be studied. Future verification and exploration are needed.

Based on our findings, hsa_circ_0000977 was upregulated in ESCC plasma and represented a meaningful diagnostic value in ESCC as a novel biomarker. Expression level is related to the pT stage and numbers of lymph nodes in patients with esophageal cancer, which may be used for the diagnosis and outcome prediction of esophageal cancer. Elevated hsa_circ_0000977 promoted cell proliferation, migration, and inhibited apoptosis in vitro ESCC cells by sponging miR‐874‐3p, whose targeted gene might be TP53INP2 or KMT2D. The enrichment results showed that the downstream gene of miR‐874‐3p was mainly involved in tumor‐related pathways such as the Hippo pathway. In conclusion, the disordered hsa_circ_0000977 expression can promote carcinogenesis in ESCC and might be a novel diagnostic biomarker to evaluate the occurrence and development of esophageal cancer.

## CONFLICT OF INTEREST

All the authors declare that there are no competing interests in this study.

## AUTHOR CONTRIBUTIONS

Ni Li and Jiacheng Wu carried out conceptualization. Ni Li and Bingchuan Hu were involved in graphics and tables. Ni Li, Linwen Zhu, and Hongna Lu carried out resources—selection and review of literature papers. Ni Li and Linwen Zhu carried out writing—original draft of the article. Jianqing Gao, Dawei Zheng carried out writing—reviewing and editing of the article.

## Supporting information

Fig S1

Table S1‐S4

## Data Availability

The datasets analyzed during the current study are available from the corresponding author upon reasonable request.
