# Supplementary figures and images for: Upregulation of hsa_circ_0000977 participates in esophageal squamous cancer progression by sponging miR‐874‐3p
Source: J Clin Lab Anal. 2022 Apr 27;36(6):e24458. doi: 10.1002/jcla.24458 (PMC9169171; doi:10.1002/jcla.24458)

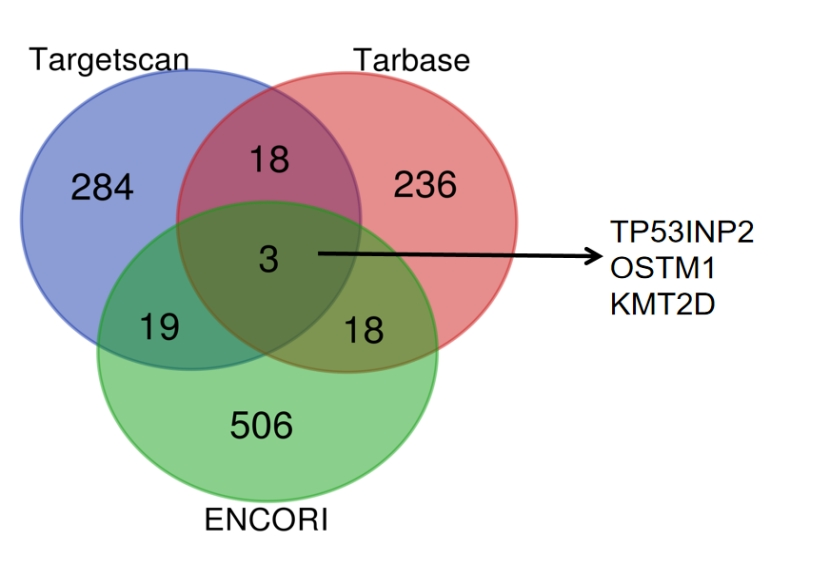

Supplement: Supplementary file 1 — Fig S1 [file JCLA-36-e24458-s001.tif]
