# Supplementary material for: Upregulation of hsa_circ_0000977 participates in esophageal squamous cancer progression by sponging miR‐874‐3p
Source: J Clin Lab Anal. 2022 Apr 27;36(6):e24458. doi: 10.1002/jcla.24458 (PMC9169171; doi:10.1002/jcla.24458)
Supplement: Supplementary file 2 — Table S1‐S4 [file JCLA-36-e24458-s002.docx]

**Supplementary Tables**

**Supplementary Table 1. Primer sequences.**

| **Primer** | **Forward primer (5’ to 3’)** | **Reverse primer (5’ to 3’)** |
| --- | --- | --- |
| hsa_circ_0000977(for qPCR) | ATGCTTCTGACGGCCAATGAA | CTGTGGCAAACAAGCCATGC |
| GAPDH (for qPCR) | AAGGTGAAGGTCGGAGTCAA | AATGAAGGGGTCATTGATGG |

**Supplementary Table 2. RNA oligonucleotide sequences.**

| **RNA oligos** | **Sequences** |
| --- | --- |
| siRNA-NC  si-hsa_circ_0000977-1 | Sense: 5’- UUCUCCGAACGUGUCACGUTT -3’  Anti-sense: 5’- ACGUGACACGUUCGGAGAATT -3’  Sense: 5’- UUACAUUCCAUUCUGAAGUTT -3’  Anti-sense: 5’-ACUUCAGAAUGGAAUGUAATT -3’ |
| si-hsa_circ_0000977-2 | Sense: 5’- ACAUUCCAUUCUGAAGUUUTT -3’  Anti-sense: 5’-AAACUUCAGAAUGGAAUGUTT -3’ |
| si-hsa_circ_0000977-3 | Sense: 5’- UCCAUUCUGAAGUUUAUAGTT -3’  Anti-sense: 5’-CUAUAAACUUCAGAAUGGATT -3’ |

**Supplementary Table 3. Summary of top 10 up regulated and top 10 down regulated circRNAs from circRNA microarray assays**

| **circRNA/Alias** | **P-value** | **FDR** | **FC (abs)** | **Regulation** | **source** |
| --- | --- | --- | --- | --- | --- |
| hsa_circ_0000977/hsa_circRNA_102619 | 0.002 | 0.040 | 46.374 | up | circBase |
| hsa_circRNA_405571 | 0.006 | 0.058 | 39.151 | up | 25070500 |
| hsa_circ_0006220/hsa_circRNA_102051 | 0.010 | 0.076 | 36.438 | up | circBase |
| hsa_circ_0043278/hsa_circRNA_102049 | 0.009 | 0.071 | 34.600 | up | circBase |
| hsa_circ_0000691/hsa_circRNA_001729 | 0.003 | 0.041 | 30.090 | up | circBase |
| hsa_circ_0000288/hsa_circRNA_404686 | 0.002 | 0.040 | 28.456 | up | 25070500 |
| hsa_circRNA_100790 | 0.029 | 0.131 | 25.419 | up | circBase |
| hsa_circ_0000367/hsa_circRNA_000367 | 0.009 | 0.072 | 23.724 | up | circBase |
| hsa_circ_0021647/hsa_circRNA_100787 | 0.035 | 0.147 | 23.416 | up | circBase |
| hsa_circRNA_006440/hsa_circRNA_006440 | 0.001 | 0.030 | 23.314 | up | circBase |
| hsa_circRNA_405965 | 0.002 | 0.040 | 23.616 | down | 25070500 |
| hsa_circ_0008389/hsa_circRNA_008389 | 0.001 | 0.027 | 19.552 | down | circBase |
| hsa_circ_0089763/hsa_circRNA_089763 | 0.006 | 0.060 | 17.978 | down | circBase |
| hsa_circ_0089762/hsa_circRNA_089762 | 0.002 | 0.039 | 17.848 | down | circBase |
| hsa_circ_0000102/hsa_circRNA_000102 | 0.002 | 0.040 | 11.289 | down | circBase |
| hsa_circ_0001714/hsa_circRNA_001714 | 0.001 | 0.027 | 11.074 | down | circBase |
| hsa_circ_0089761/hsa_circRNA_089761 | 0.036 | 0.148 | 10.853 | down | circBase |
| hsa_circ_0007326/hsa_circRNA_007326 | 0.000 | 0.018 | 9.916 | down | circBase |
| hsa_circ_0001549/hsa_circRNA_000424 | 0.000 | 0.020 | 7.868 | down | circBase |
| hsa_circ_0005133/hsa_circRNA_005133 | 0.000 | 0.019 | 7.491 | down | circBase |

**Supplementary Table 4. Summary of miR-874-3p predicted target genes GO / KEGG enrichment analysis**

| ONTOLOGY | ID | Description | GeneRatio | BgRatio | pvalue |
| --- | --- | --- | --- | --- | --- |
| BP | GO:0030099 | myeloid cell differentiation | 47/972 | 416/18670 | 5.00e-07 |
| BP | GO:0031952 | regulation of protein autophosphorylation | 13/972 | 47/18670 | 5.13e-07 |
| BP | GO:2000027 | regulation of animal organ morphogenesis | 33/972 | 253/18670 | 1.12e-06 |
| BP | GO:0048588 | developmental cell growth | 30/972 | 234/18670 | 4.83e-06 |
| BP | GO:0046777 | protein autophosphorylation | 30/972 | 235/18670 | 5.27e-06 |
| CC | GO:0035770 | ribonucleoprotein granule | 31/990 | 223/19717 | 2.72e-07 |
| CC | GO:0036464 | cytoplasmic ribonucleoprotein granule | 28/990 | 212/19717 | 2.84e-06 |
| CC | GO:0000932 | P-body | 16/990 | 84/19717 | 3.69e-06 |
| CC | GO:0032279 | asymmetric synapse | 34/990 | 328/19717 | 5.31e-05 |
| CC | GO:0031252 | cell leading edge | 39/990 | 403/19717 | 7.12e-05 |
| MF | GO:0019903 | protein phosphatase binding | 22/973 | 140/17697 | 7.90e-06 |
| MF | GO:0004674 | protein serine/threonine kinase activity | 45/973 | 439/17697 | 4.33e-05 |
| MF | GO:0003714 | transcription corepressor activity | 29/973 | 238/17697 | 4.95e-05 |
| MF | GO:0003725 | double-stranded RNA binding | 14/973 | 75/17697 | 5.08e-05 |
| MF | GO:0005154 | epidermal growth factor receptor binding | 9/973 | 33/17697 | 5.15e-05 |
| KEGG | hsa05223 | Non-small cell lung cancer | 11/428 | 72/8076 | 0.001 |
| KEGG | hsa04218 | Cellular senescence | 18/428 | 156/8076 | 0.001 |
| KEGG | hsa04390 | Hippo signaling pathway | 18/428 | 157/8076 | 0.002 |
| KEGG | hsa05220 | Chronic myeloid leukemia | 11/428 | 76/8076 | 0.002 |
| KEGG | hsa05226 | Gastric cancer | 17/428 | 149/8076 | 0.002 |
